# Supplementary material for: MFSD1 with its accessory subunit GLMP functions as a general dipeptide uniporter in lysosomes
Source: Nat Cell Biol. 2024 Jun 5;26(7):1047–61. doi: 10.1038/s41556-024-01436-5 (PMC11252000; doi:10.1038/s41556-024-01436-5)
Supplement: Supplementary file 1 — Supplementary Figs. 1–3 and captions for Tables 1–5; caption for Video 1. [file 41556_2024_1436_MOESM1_ESM.pdf]

nature cell biology

Supplementary information https://doi.org/10.1038/s41556-024-01436-5

MFSD1 with its accessory subunit GLMP functions as a general dipeptide uniporter in lysosomes

In the format provided by the authors and unedited

Supplementary Information

Table-of-contents

Supplementary Figures 1 - 3.

Supplementary Figure 1. Multiple sequence alignment of MFSD1 with other MFSD1 homologs.

Supplementary Figure 2. Comparison of Cryo-EM structure of MFSD1 to AlphaFold2 models in different conformations

Supplementary Figure 3. Multiple sequence alignment of GLMP with other GLMP homologs.

Supplementary Tables 1 - 5.

Supplemental Table 1. Metabolomics raw data.

Supplemental Table 2. List of compounds for nanoDSF screening.

Supplemental Table 3. Selected ions monitored (SIM) and multiple reaction monitoring (MRM) for LC-MS/MS analysis.

Supplemental Table 4. Cryo-EM data collection, refinement and validation statistics.

Supplemental Table 5. Description of the molecular dynamics simulations.

Supplementary Movie 1.

Legend Movie S1. Conformational changes of MFSD1 during the transport cycle.

Supplemental Figure 1

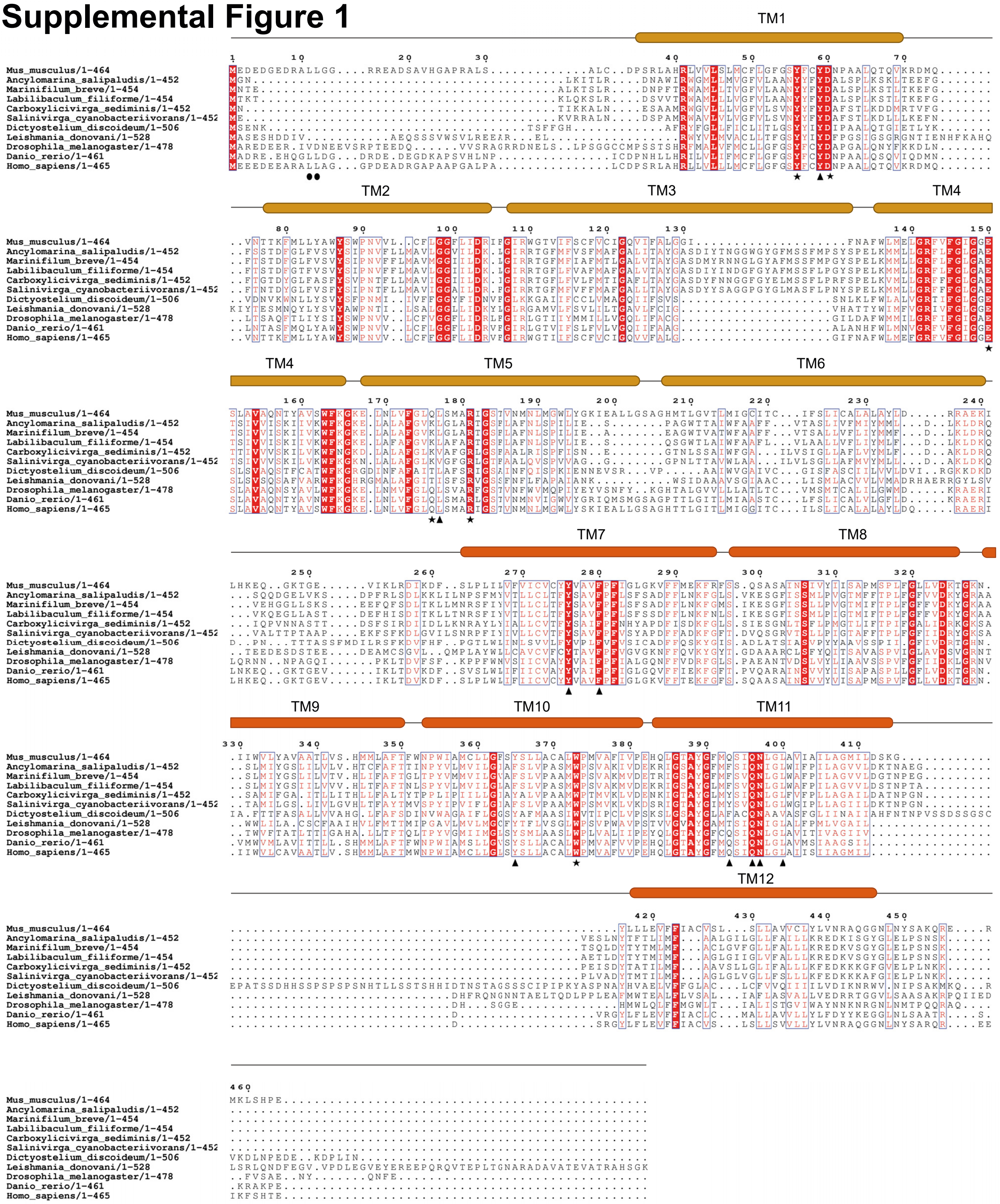

Supplementary Figure 1. Multiple sequence alignment of MFSD1 with other MFSD1 homologs. Sequence alignment of MFSD1 from *Mus musculus* (accession code: Q9DC37), *Ancylomarina salipaludis* (accession code: WP_129252052.1), *Marinifilum breve* (accession code: WP_165836040.1), *Labilibaculum filiforme* (accession code: WP_180335631.1), *Carboxylicivirga sediminis* (accession code: WP_212192738.1), *Salinivirga cyanobacteriivorans* (accession code: WP_057952039.1), *Dictyostelium discoideum* (accession code: XP_636334.1), *Leishmania donovani* (accession code: XP_003862116.1), *Drosophila melanogaster* (accession code: NP_001261505.1), *Danio rerio* (accession code: Q32LQ6) and *Homo sapiens* (accession code: Q9H3U5) using ClustalO. Conserved residues are highlighted in red letters, whereas highly conserved residues are shown with a red background. (●) dileucine motif, (▲) binding site residues, (★) binding site residues used for mutational studies. Above the sequence, the transmembrane helices are highlighted based on the determined structure, colored in dark yellow for the N-domain and orange for the C-domain.

Supplemental Figure 2

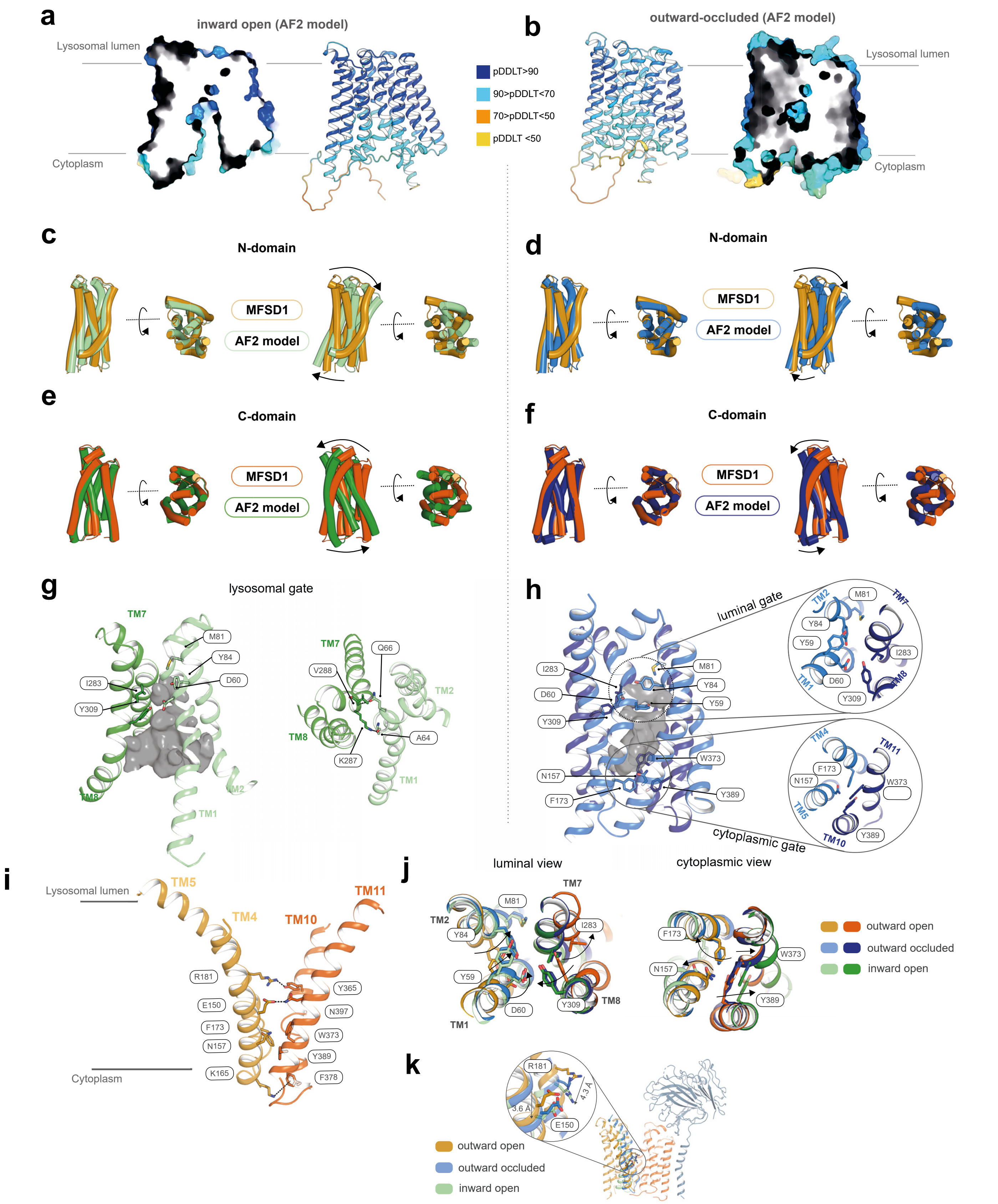

Supplementary Figure 2. Comparison of Cryo-EM structure of MFSD1 to AlphaFold2 models in different conformations. (a) AlphaFold2 (AF) prediction of MFSD1 in an inward open conformation colored by its pDDLT score. **(b)** AF model of the outward occluded conformation of MFSD1 colored by its pDDLT score. **(c)** Superposition of N-domains of MFSD1 Cryo-EM model (yellow) and inward-open AF model (pale green). The superpositions are shown either by

aligning the N-domains only (left panel) or the entire model of MFSD1 (right panels). Observed changes are indicated by arrows. **(d)** Superposition of N-domains of MFSD1 Cryo-EM model (yellow) and the predicted outward-occluded AF model (pale blue). The superpositions represent the alignments of the N-domains only (left panel) or the complete model of MFSD1 (right panels). **(e)** Superposition of C-domains of MFSD1 cryoEM model (yellow) and inward-open AF model (forest green). The superpositions are shown either by aligning the N-domains (left panel) only or the entire model of MFSD1 (right panels). **(f)** Superposition of C-domains of MFSD1 Cryo-EM model (yellow) and outward-occluded AF model (sky blue). In the left panel, only the C-domains (left panel) were aligned, while the right panel represents the alignment of the entire transporter between the two states. **(g)** Illustration of the luminal gate of the inward-open AF model. Crucial residues involved in gate formation are indicated. **(h)** Luminal and cytoplasmic gates (including the close-up view) of outward-occluded AF model of MFSD1. Residues forming the gates are indicated. **(i)** Cytoplasmic gate and inter-bundle interactions of the outward-open state Cryo-EM model of MFSD1. **(j)** Cartoon representation of the conformational transitions of the gating residues of the outward-open Cryo-EM MFSD1 model and the two AF-predicted conformations viewed from the luminal and cytoplasmic side. The key residues forming the cytoplasmic and luminal gates are shown for each conformation. Color codes for the respective conformations are indicated. Arrows highlight the movement of each gating residue during the transition from inward to outward open. (**k**) Movement of residues E150 and R181, residing in the substrate-binding site within the N-domain (yellow) of MFSD1, during the transport cycle from outward-open (N-domain:yellow and C-domain:orange) to inward-open conformation (N-domain:green) highlighted with arrows to indicate the direction of the movement.

Supplemental Figure 3

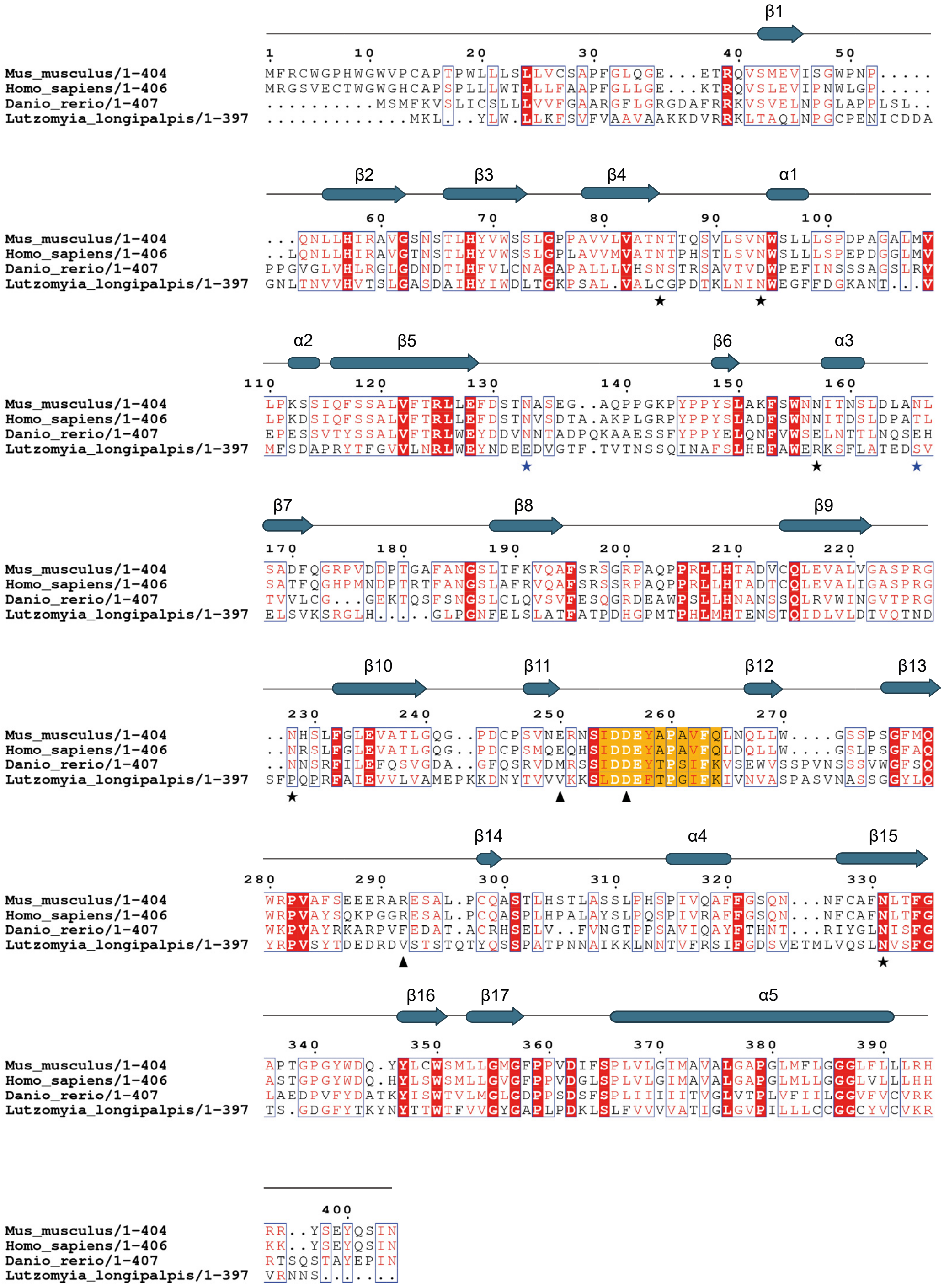

Supplementary Figure 3. Multiple sequence alignment of GLMP with other GLMP homologs. Sequence alignment of GLMP from *Mus musculus* (accession code: Q9JHJ3), *Danio rerio* (accession code: Q66HW4), *Lutzomyia longipalpis* (accession code: A0A7G3AQ10), and *Homo sapiens* (accession code: Q8WWB7) using ClustalO. Conserved residues are highlighted in red font, whereas highly conserved residues are shown with red background. Residues with a (★) represent identified glycosylation sites of GLMP in the Cryo-EM structure, whereas (★) label additional glycosylation sites as listed in UniProt. Residues highlighted with (▲) were mutated to test for the rescue of MFSD1 by GLMP. Colored in yellow is the loop region that was exchanged for Ala-Ala-Ala-Ala-Ala to test for MFSD1 rescue by GLMP reexpression in this study. Secondary structure elements of GLMP are shown above the alignment depicted by beta sheets (blue arrows) and alpha helices (blue tubes).
