# Supplementary material for: MFSD1 with its accessory subunit GLMP functions as a general dipeptide uniporter in lysosomes
Source: Nat Cell Biol. 2024 Jun 5;26(7):1047–61. doi: 10.1038/s41556-024-01436-5 (PMC11252000; doi:10.1038/s41556-024-01436-5)
Supplement: Supplementary file 2 — Reporting Summary [file 41556_2024_1436_MOESM2_ESM.pdf]

nature portfolio

Corresponding author(s): Markus Damme, Christian Löw, Bruno Gasnier

Last updated by author(s): Apr 27, 2024

Reporting Summary

Nature Portfolio wishes to improve the reproducibility of the work that we publish. This form provides structure for consistency and transparency in reporting. For further information on Nature Portfolio policies, see our Editorial Policies and the Editorial Policy Checklist.

Statistics

For all statistical analyses, confirm that the following items are present in the figure legend, table legend, main text, or Methods section.

n/a	Confirmed	
☐	☒	The exact sample size (*n*) for each experimental group/condition, given as a discrete number and unit of measurement
☐	☒	A statement on whether measurements were taken from distinct samples or whether the same sample was measured repeatedly
☐	☒	The statistical test(s) used AND whether they are one- or two-sided<br>*Only common tests should be described solely by name; describe more complex techniques in the Methods section.*
☒	☐	A description of all covariates tested
☒	☐	A description of any assumptions or corrections, such as tests of normality and adjustment for multiple comparisons
☒	☐	A full description of the statistical parameters including central tendency (e.g. means) or other basic estimates (e.g. regression coefficient) AND variation (e.g. standard deviation) or associated estimates of uncertainty (e.g. confidence intervals)
☐	☒	For null hypothesis testing, the test statistic (e.g. *F*, *t*, *r*) with confidence intervals, effect sizes, degrees of freedom and *P* value noted<br>*Give P values as exact values whenever suitable.*
☒	☐	For Bayesian analysis, information on the choice of priors and Markov chain Monte Carlo settings
☒	☐	For hierarchical and complex designs, identification of the appropriate level for tests and full reporting of outcomes
☐	☒	Estimates of effect sizes (e.g. Cohen's *d*, Pearson's *r*), indicating how they were calculated

Our web collection on statistics for biologists contains articles on many of the points above.

Software and code

Policy information about availability of computer code

Data collection	The Cryo-EM data was collected using EPU2.8.0.1256REL (Thermo Fisher Scientific);<br>The Thermal Stability data was collected using PR.ThermControl v.2.1.2 (Nanotemper);<br>The fluorescent data for liposome-based assay was collected using i-control™ (Tecan);<br>Multi-target LC-MS/MS analysis of oocyte extracts was performed using the LabSolutions v 5.118 software (Shimadzu Corporation)<br>Electrophysiological recordings were made with Clampex V. 11.2.2.17 (Molecular Devices);<br>The confocal immunofluorescence microscopy data was collected using a LSM980 + AiryScan (Zeiss) with Zen Blue software V. 3.2<br>Molecular Dynamics simulations were performed using GROMACS 2021.3.
Data analysis	The Cryo-EM data was processed using CryoSPARCv3. Refinement and validation: Isolde v1.6, Phenix v.1.20.1; Coot v.0.9.8.1; ChimeraX 1.3; PyMOL v2.5.5; MolProbity 4.2<br>For sequence alignments ClustalOmega (no version) and visualization ESPript 3.<br>Structure modeling was performed using AlphaFold2 and AlphaFold2 Multimer;<br>Thermal Stability data was analyzed using PR.ThermControl v.2.1.2 (Nanotemper), MoltenProt v0.2.1; Visualized using Graphpad Prism v.9.5.1<br>Targeted LC-MS/MS chromatograms were analysed using LabSolutions v 5.118 (Shimazu)<br>Electrophysiological data were analyzed with Clampfit v. 11.2.2.17 (Molecular Devices)<br>ImageJ 1.52 was used for adjustment of images<br>Graphpad Prism v.9.5.1 was used for general statistics

For manuscripts utilizing custom algorithms or software that are central to the research but not yet described in published literature, software must be made available to editors and reviewers. We strongly encourage code deposition in a community repository (e.g. GitHub). See the Nature Portfolio guidelines for submitting code & software for further information.

Data

Policy information about availability of data

All manuscripts must include a data availability statement. This statement should provide the following information, where applicable:

- Accession codes, unique identifiers, or web links for publicly available datasets
- A description of any restrictions on data availability
- For clinical datasets or third party data, please ensure that the statement adheres to our policy

The EM data and fitted models for GLMP-MFSD1 have been deposited in the Electron Microscopy Data Bank under accession code EMD-19005 and the PDB under accession code 8R8Q. Raw data used for data plotting are available as a supplementary table. The crystal structure of GLMP used for comparative analysis in this study can be found in the PDB under accession code 6NYQ AlphaFold2 predictions of MFSD1 as well as the models of MFSD1 and GLMP-MFSD1 after 500 ns of molecular dynamics simulations and metabolomics raw data were deposited to Zenodo (Alphafold models: 10.5281/zenodo.10276738; MFSD1 apo/with ligands in initial poses and after 500 ns MD: 10.5281/zenodo.10276760;). All protein sequences used in this study are publicly available at Uniprot (https://www.uniprot.org/). The metabolomics data are available at 10.5281/zenodo.10839783. Source data have been provided in Source Data. All other data supporting the findings of this study are available from the corresponding author on reasonable request.

Research involving human participants, their data, or biological material

Policy information about studies with human participants or human data. See also policy information about sex, gender (identity/presentation), and sexual orientation and race, ethnicity and racism.

Reporting on sex and gender	N/A
Reporting on race, ethnicity, or other socially relevant groupings	N/A
Population characteristics	N/A
Recruitment	N/A
Ethics oversight	N/A

Note that full information on the approval of the study protocol must also be provided in the manuscript.

Field-specific reporting

Please select the one below that is the best fit for your research. If you are not sure, read the appropriate sections before making your selection.

☒ Life sciences ☐ Behavioural & social sciences ☐ Ecological, evolutionary & environmental sciences

For a reference copy of the document with all sections, see nature.com/documents/nr-reporting-summary-flat.pdf

Life sciences study design

All studies must disclose on these points even when the disclosure is negative.

Sample size	No sample-size calculation was performed. The sample size for each experiment is included in the respective figure legend and statistical methods were used to calculate standard deviation as noted in figure legends. Experiments were performed with at least n = 3; n-numbers are provided in the figures/figure legends. Sample size was determined based on similar studies in this field and our own experience obtaining consistent data throughout replicating the experiments.
Data exclusions	Data were only excluded if obvious technical problems occurred during the experiments. Generally, no data was excluded.
Replication	Experiments were performed with at least n = 3. For each representative image/dataset, at least 2 other independent experiments were successfully repeated.
Randomization	Samples were not randomized for this study because all experiments were internally controlled.
Blinding	Blinding was not performed as is not applicable to the study for many experiments (purification of the recombinant proteins, cryo-EM, nano-DSF experiments with recombinant proteins, oocyte overexpressing MFSD1/GLMP).

Reporting for specific materials, systems and methods

We require information from authors about some types of materials, experimental systems and methods used in many studies. Here, indicate whether each material, system or method listed is relevant to your study. If you are not sure if a list item applies to your research, read the appropriate section before selecting a response.

Materials & experimental systems

n/a	Involved in the study
☐	☒ Antibodies
☐	☒ Eukaryotic cell lines
☒	☐ Palaeontology and archaeology
☐	☒ Animals and other organisms
☒	☐ Clinical data
☒	☐ Dual use research of concern
☒	☐ Plants

Methods

n/a	Involved in the study
☒	☐ ChIP-seq
☒	☐ Flow cytometry
☒	☐ MRI-based neuroimaging

Antibodies

Antibodies used	LAMP1 clone 1D4B (purified rat monoclonal, Developmental Studies Hybridoma Bank); LAMP1 clone 1D4B (purified rat monoclonal, conjugated to AlexaFluor 647, #121609, BioLegend); HA clone 3F10 (rat monoclonal, ROAHAHA/11867423001; Sigma-Aldrich / Merck),); HA clone 3F10 (rat monoclonal, conjugated to FITC, 11988506001; Sigma-Aldrich / Merck), GFP (11814460001, mouse monoclonal, Roche Molecular Biochemicals), mKate2 (rabbit polyclonal, TA150072, Origene), KDEL (clone 10C3, mouse monoclonal, ADI-SPA-827-D, Enzo Life Sciences), Cox IV (rabbit polyclonal, ab16056, Abcam), Golgin 97 (clone CDF4, mouse monoclonal, A-21270, Thermo Scientific Fisher). The antibody against cathepsin D (CTSD) was custom-made against a synthetic peptide (CKSDQSKARGIKVEKQIFGEATKQP) and immunization of rabbits, followed by affinity purification against the immunization peptide. The custom-made MFSD1- and GLMP-specific rabbit polyclonal antibodies were described before (Massa Lopez et al., 2019 / PMID: 31661432).<br>Secondary antibodies: HRP-coupled goat anti rat (112-035-143, Dianova); goat anti mouse (115-035-146, Dianova), goat anti rabbit (111-035-144, Dianova)
Validation	Well established commercial antibodies were used throughout the study. Whenever possible, monoclonal antibodies were used. MFSD1, GLMP and CTSD specific antibodies were in house knockout validated (MFSD1/GLMP: PMID: 31661432; CTSD: unpublished). LAMP1 1D4B mAb is knockout validated (PMID: 10212251). Tag-specific monoclonal antibodies (3F10 / HA; GFP; mKate2) were validated upon the overexpression of tagged proteins with the corresponding tag; Golgin 97 (clone CDF4) is well-established with >100 citations. Cox IV is well-established with >200 citations. KDEL (clone 10C3) is well-established with >40 citations. Additional antibody validation can be found on the manufacturer's website.

Eukaryotic cell lines

Policy information about cell lines and Sex and Gender in Research

Cell line source(s)	Expi293F Thermo Fisher (Cat. number: A14527), Mouse embryonic fibroblasts (MEF); Winnie Eskild lab
Authentication	None of the cell lines used were authenticated.
Mycoplasma contamination	We confirmed that Expi293F cells were tested negative for mycoplasma contamination.
Commonly misidentified lines<br>(See ICLAC register)	No commonly misidentified cell line was used

Animals and other research organisms

Policy information about studies involving animals; ARRIVE guidelines recommended for reporting animal research, and Sex and Gender in Research

Laboratory animals	Mice: C57Bl/6N-Mfsd1tm1dHhtg/Damme; age 6 months; Mice were housed under standard laboratory conditions with a 12-hour light/dark cycle and constant room temperature and humidity. Food and water were available ad libitum.<br>Xenopus laevis oocytes. Female Xenopus frogs (age: 6- 10 years) were used for the production of oocytes.
Wild animals	No wild animals were used.
Reporting on sex	Female and male mice were used for the study and the gender was not considered in the study design.
Field-collected samples	The study did not involve samples collected from the field.
Ethics oversight	Mice: ethical agreement Ministerium für Energiewende, Klimaschutz, Umwelt und Natur V242-13648/2018<br>Xenopus: ethical agreement Ministère de l'Enseignement Supérieur et de la Recherche, France APAFiS #14316-2017112311304463 v4

Note that full information on the approval of the study protocol must also be provided in the manuscript.

Plants

Seed stocks	N/A
Novel plant genotypes	N/A
Authentication	N/A
