# Supplementary material for: MFSD1 with its accessory subunit GLMP functions as a general dipeptide uniporter in lysosomes
Source: Nat Cell Biol. 2024 Jun 5;26(7):1047–61. doi: 10.1038/s41556-024-01436-5 (PMC11252000; doi:10.1038/s41556-024-01436-5)

Source Data Figure 1

Figure 1b

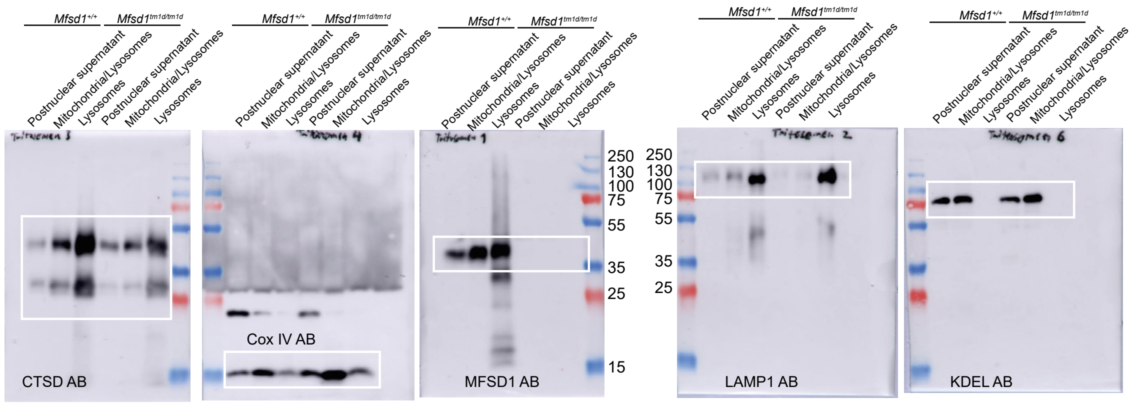

Figure 1F

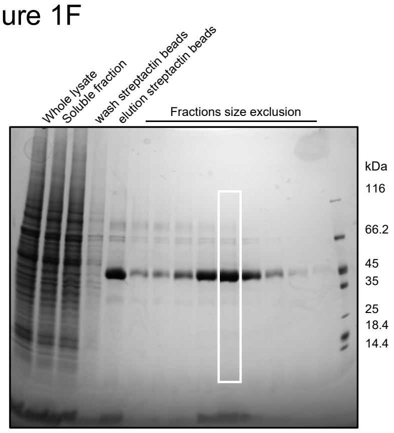

Source Data Figure 3

Figure 3a

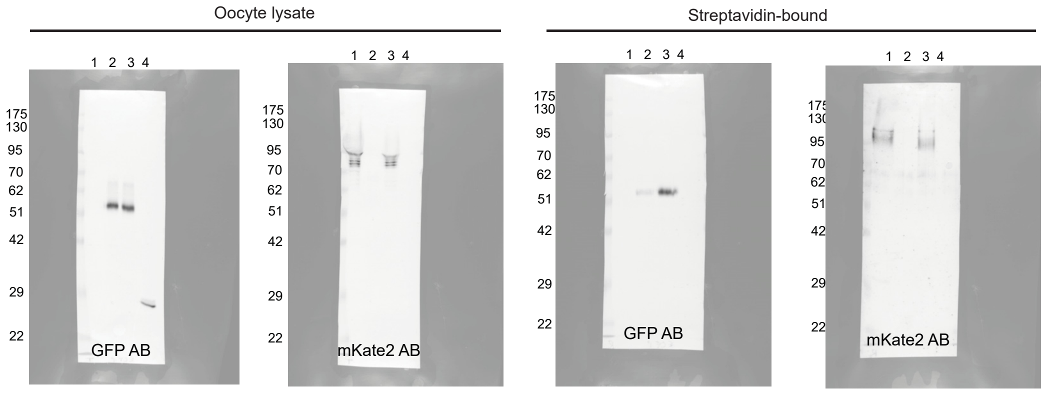

Lanes:
1 = GLMP-Y400A-mKate2
2 = MFSD1-L11A/L12A-EGFP
3 = 1+2 co-expression
4 = EGFP

Source Data Figure 4

Figure 4b

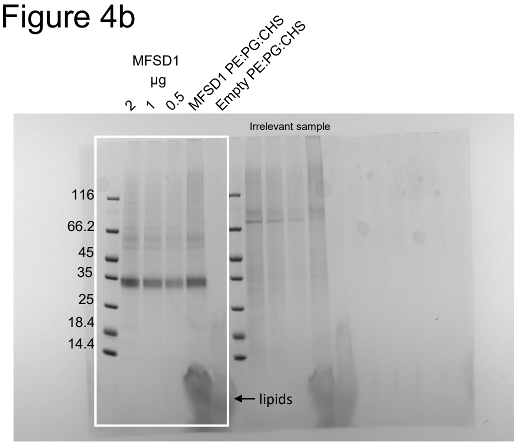

Source Data Extended Figure 5

Extended Figure 5a

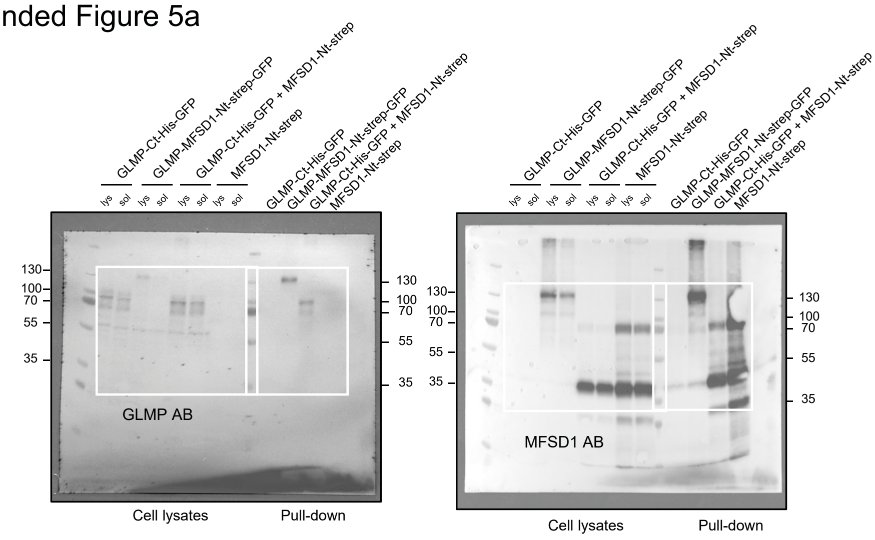

Extended Figure 5b & 5e

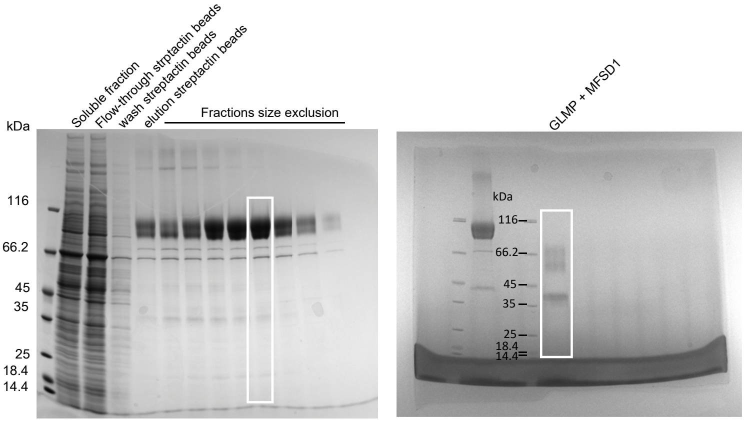

Source Data Extended Figure 10

Extended Figure 10b

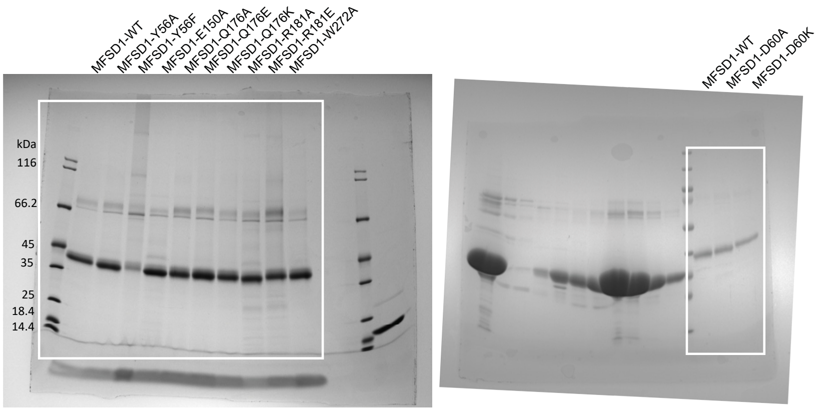

Supplement: Supplementary file 7 — Unprocessed western blots and/or gels. [file 41556_2024_1436_MOESM7_ESM.pdf]
